# Fluid assessment, fluid balance, and fluid overload in sick children: a report from the Pediatric Acute Disease Quality Initiative (ADQI) conference

**DOI:** 10.1007/s00467-023-06156-w

**Published:** 2023-11-07

**Authors:** David T. Selewski, Matthew F. Barhight, Erica C. Bjornstad, Zaccaria Ricci, Marcelo de Sousa Tavares, Ayse Akcan-Arikan, Stuart L. Goldstein, Rajit Basu, Sean M. Bagshaw, on behalf of the Pediatric the Acute Disease Quality Initiative (ADQI) Consensus Committee Members, Rashid Alobaidi, David J. Askenazi, Erin Barreto, Benan Bayrakci, O. N. Ray Bignall, Patrick Brophy, Jennifer Charlton, Rahul Chanchlani, Andrea L. Conroy, Akash Deep, Prasad Devarajan, Kristin Dolan, Dana Fuhrman, Katja M. Gist, Stephen M. Gorga, Jason H. Greenberg, Denise Hasson, Emma Heydari, Arpana Iyengar, Jennifer Jetton, Catherine Krawczeski, Leslie Meigs, Shina Menon, Catherine Morgan, Jolyn Morgan, Theresa Mottes, Tara Neumayr, Danielle Soranno, Natalja Stanski, Michelle Starr, Scott M. Sutherland, Jordan Symons, Molly Vega, Michael Zappitelli, Claudio Ronco, Ravindra L. Mehta, John Kellum, Marlies Ostermann

**Affiliations:** 1 Division of Nephrology, Department of Pediatrics, Medical University of South Carolina, Charleston, SC USA; 2 Division of Critical Care, Ann & Robert H. Lurie Children’s Hospital, Chicago, IL USA; 3 Division of Pediatric Nephrology, University of Alabama at Birmingham, Birmingham, AL USA; 4 Department of Emergency and Intensive Care, Pediatric Intensive Care Unit, Azienda Ospedaliero Universitaria Meyer, Florence, Italy; 5 Department of Health Science, University of Florence, Florence, Italy; 6 Pediatric Nephrology Unit, Nephrology Center of Santa Casa de Belo Horizonte, Belo Horizonte, Minas Gerais Brazil; 7 Division of Nephrology, Department of Pediatrics, Baylor College of Medicine, Texas Children’s Hospital, Houston, TX USA; 8 Division of Critical Care Medicine, Department of Pediatrics, Baylor College of Medicine, Texas Children’s Hospital, Houston, TX USA; 9 Center for Acute Care Nephrology, Cincinnati Children’s Hospital Medical Center, Cincinnati, OH USA; 10 Department of Critical Care Medicine, Faculty of Medicine and Dentistry, University of Alberta and Alberta Health Services, Edmonton, AB Canada

**Keywords:** Acute kidney injury, Fluid balance, Fluid overload, Pediatric, Neonatal, Continuous kidney replacement therapy

## Abstract

**Background:**

The impact of disorders of fluid balance, including the pathologic state of fluid overload in sick children has become increasingly apparent. With this understanding, there has been a shift from application of absolute thresholds of fluid accumulation to an appreciation of the intricacies of fluid balance, including the impact of timing, trajectory, and disease pathophysiology.

**Methods:**

The 26th Acute Disease Quality Initiative was the first to be exclusively dedicated to pediatric and neonatal acute kidney injury (pADQI). As part of the consensus panel, a multidisciplinary working group dedicated to fluid balance, fluid accumulation, and fluid overload was created. Through a search, review, and appraisal of the literature, summative consensus statements, along with identification of knowledge gaps and recommendations for clinical practice and research were developed.

**Conclusions:**

The 26th pADQI conference proposed harmonized terminology for fluid balance and for describing a pathologic state of fluid overload for clinical practice and research. Recommendations include that the terms *daily fluid balance*, *cumulative fluid balance*, and *percent cumulative fluid balance* be utilized to describe the fluid status of sick children. The term *fluid overload* is to be preserved for describing a pathologic state of positive fluid balance associated with adverse events. Several recommendations for research were proposed including focused validation of the definition of fluid balance, fluid overload, and proposed methodologic approaches and endpoints for clinical trials.

**Supplementary Information:**

The online version contains supplementary material available at 10.1007/s00467-023-06156-w.

## Introduction

In recent years, the deleterious impact of fluid overload in critically ill patients across the age spectrum has become clear [1–20]. Evidence first described in children has led the field in highlighting the adverse outcomes associated with excessive fluid accumulation in sick children. The association between greater positive fluid balance at the time of initiation of continuous kidney replacement therapy (CKRT) and increased mortality was first described in 2001 [8]. This association has been demonstrated repeatedly across a spectrum of neonatal and pediatric populations, including those who are critically ill, receiving mechanical ventilation, on extracorporeal life support, and undergoing congenital heart surgery. Thus, the importance of documenting and monitoring fluid balance and for complications attributed to fluid accumulation is vital to avoiding adverse events and improving outcomes in sick children.

Careful examination of the concept of “fluid overload” in children reveals several gaps in our understanding of the impact of fluid accumulation. In early studies, the term *fluid overload* was used to simply describe positive fluid accumulation in children at the time of CKRT initiation [7–9, 12, 17, 20, 21]. In these studies, fluid accumulation likely represented a pathologic state was equated with fluid overload, and children by extension were perceived to benefit from intervention with CKRT. Since those initial studies, the terminology in the literature describing fluid accumulation has largely used the term *fluid overload* to equate with a state of positive fluid balance. However, use of the term *fluid overload* in this way introduces bias by the inherent assumption that all fluid accumulation is detrimental, and by extension fluid removal or “negative fluid overload” is good. Furthermore, the methodology utilized to calculate fluid balance in these studies varied [8, 12–14, 17, 20]. To advance our understanding and generate new knowledge, a standardization of terminology and methods for reporting fluid balance is necessary.

Fluid accumulation represents an attractive target for intervention in sick children. Several strategies to prevent or mitigate a state of *fluid overload* have been proposed [22]. Prior to widespread implementation of such strategies, however, it is vital to appraise the source and strength of existing evidence to support the association between positive fluid balance and outcomes in sick children. The current literature has significant limitations, including being small, single center, and observational (often retrospective) in design, along with a lack of standardized reporting on measures of fluid balance (definition, timing, epidemiology) across studies [23–25]. Rigorous observational studies and randomized trials evaluating prospective interventions and strategies to manage fluid balance and accumulation in sick children are needed.

Acknowledging the importance of fluid accumulation and disorders of fluid balance, the meeting chairs of the first ADQI dedicated to neonates and pediatric patients (the 26th ADQI) convened a diverse expert multidisciplinary working group dedicated to fluid balance, fluid accumulation, and fluid overload. We herein summarize the consensus to describe in detail: definitions and epidemiology of disorders of fluid balance, targets for intervention, and endpoints for clinical trials.

## Methods

The methodology utilized for ADQI meetings has been developed iteratively over the last two decades [26]. The aim of ADQI meetings is to provide expert-based statements, supported by evidence where applicable, and interpretation of current knowledge for use in clinical care and research endeavors. In addition, ADQI aims to identify evidence and knowledge-to-care gaps to establish future research priorities. The 26th ADQI consensus meeting included physicians and scientists (adult and pediatric nephrology and critical care, pediatric cardiology), nurse practitioners, nurse educators, clinical pharmacists, critical care dieticians, health services researchers, and patient and family advocates for a 3-day meeting held in Napa Valley, CA on November 11–14, 2021.

The preparation began over a year prior to the in-person meeting with a detailed literature review (2001–2021), topic elicitation, question development, and proposed statements. The workgroup identified several themes around “fluid balance and fluid overload” to generate concrete questions for the in-person meeting which were iteratively refined though consensus during sequential plenary sessions.

## Question 1: What defines fluid balance in sick children?

### Statement: Fluid balance is the difference between total input and output that can be expressed as “daily” and “cumulative” over a defined duration of time (Table 1)

**Table 1:** Definitions of fluid balance [27]

Terminology	Measurement/equation	Duration
Cumulative fluid input and output methodology [2, 8, 9, 15, 20]		
Daily fluid balance	Fluid intake (L) − fluid output (L)	Over 24 h
Cumulative fluid balance	∑Fluid intake (L) − fluid output (L)	Over a defined period of time
Percent cumulative fluid balance	$\sum \frac{[\text{Fluid intake}\;\left(\text{L}\right)-\text{fluid output }\;\left(\mathrm{L}\right)]* 100\mathrm{\%}}{\text{anchor weight (kg)}}$	Over a defined period of time
Weight-based methodology [4, 13, 17, 28, 29]		
Daily fluid balance	Current weight (kg) − weight from previous day (kg)	Over 24 h
Cumulative fluid balance	Current weight (kg) − anchor weight (kg)	Over a defined period of time
Percent cumulative fluid balance	$\frac{[\text{Current weight }\left(\mathrm{kg}\right)-\text{ anchor weight }\left(\mathrm{kg}\right)]* 100\mathrm{\%}}{\text{anchor weight (kg)}}$	Over a defined period of time

-Fluid intake: this includes all input of intravenous fluids, blood products, enteral feeds, and medications. This does not distinguish enteral vs. intravenous input

-Fluid output: this includes all recorded output including urine, drains, dressings, stool. This does not account for insensible losses

-Anchor weight: the most common anchor weight used in the literature is ICU admission weight. The anchor weight can be customized to specific populations and duration of hospitalization (e.g., pre-operative weight in children undergoing scheduled surgery, birthweight in neonates during the first 2 postnatal weeks, hospital admission weight for those never admitted to ICUs or facilities without ICUs, outpatient weight prior to hospitalization)

Many terms have been used interchangeably to describe the impact of fluid on outcomes in sick children, including but not limited to “fluid balance,” “fluid accumulation,” and “fluid overload.” Identifying consensus terminology is critical to advance our understanding of the impact of fluid management on outcomes across populations. Such definitions will guide the development of clinical practice guidelines, epidemiological surveillance, clinical trials, and ultimately improved care for sick children and neonates.

The concept of fluid balance is at the heart of describing fluid accumulation in any population. Fluid balance is a measure of the intake and output for a given duration. This is classically expressed over a period of time as daily fluid balance (24-h) or cumulative fluid balance (since admission, previous number of days, etc.). The cumulative fluid balance is often expressed as a percent corrected for a pre-specified *anchor weight*. We propose utilizing the terms *daily fluid balance*, *cumulative fluid balance*, and *percent cumulative fluid balance* to describe the fluid status objectively in sick children for the purpose of clinical care and research (Table 1).

The two methodologies commonly utilized in the literature to describe fluid balance include cumulative fluid input and output and weight-based methodology (Table 1). The “cumulative fluid input and output methodology” has been adopted since the aforementioned 2001 study that first described the impact of “fluid overload” on outcomes in critically ill children initiating CKRT [8, 11, 15, 20, 27, 30]. This is the most frequently used methodology of quantifying fluid accumulation in the literature.

With this methodology, practitioners must be able to accurately measure all inputs (intravenous fluids, blood products, enteral feeds, intravenous flushes, etc.) and all outputs (urine, drains, dressings, stool, etc.) over a given time period. In the absence of a Foley catheter, urine output is often calculated utilizing change in diaper weight or measured voids which introduces potential limitations. Inaccuracies or missing input/output measurements are carried forward in all subsequent fluid balance calculations, propagating any potential inaccuracy. Enteral and intravenous fluid (IVF) contributions to input are generally treated as equivalent. Furthermore, this methodology does not account for insensible losses or potential insensible gains (humidified ventilatory circuits).

The second methodology to describe fluid balance utilizes a weight-based approach [4, 14, 16–18] as a change in patient weight from an anchor weight. The ability to reliably measure weight in a consistent and safe matter is crucial for application of this approach, which frequently requires set protocols to account for potential barriers such as mechanical ventilation, extracorporeal membrane oxygenation (ECMO), and use of bed weights. In this context, a reliable weight does not necessarily reflect the patient’s “true weight,” but instead a weight that is measured using a consistent method from day to day. This approach removes the inherent inaccuracies of accounting for daily input and output and should theoretically capture insensible and other losses. This would also enable compensation for missed or inaccurate daily measures of fluid input and output in the calculation of cumulative fluid balance.

For either method, a weight is needed in the denominator to understand the percent change in fluid balance by serving as a baseline for the equation or the *anchor weight*. Anchor weights used to describe percent change in fluid balance in the literature include intensive care unit (ICU) admission weight, hospital admission weight, estimated dry weight, pre-operative weight, and birthweight in neonates [4, 7–9, 12, 14, 17, 28, 31]. The ICU admission weight is the weight most commonly utilized as the anchor weight for fluid balance calculations in the literature*.* This practice likely reflects the fact that the ICU admission weight often is the first recorded weight available to clinicians. Less commonly reported in the literature is the use of hospital admission weight, which may reflect the weight in the emergency department, ICU admission weight, or ward admission weight. This may risk introducing bias due to the lack of standardization of weighing practices across different areas. The admission weight represents patients in a variety of fluid balance states ranging from hypo- to hypervolemic. Finally, some reports have used the physician estimate of “dry weight” as the baseline for weight correction. However, clinicians do not reliably predict dry weight, recognize fluid overload clinically, and the dry weight implies a healthy state with muscle mass that may significantly change over the course of a critical illness [31]. Therefore, no clear gold standard exists against which to systematically compare different approaches, highlighting a knowledge gap requiring targeted study.

A vital point for defining fluid balance is the concept of *euvolemia*, commonly referred to clinically as an estimated healthy dry weight. In considering fluid balance, we must acknowledge some of the inherent assumptions commonly utilized in the literature and their weaknesses. In particular, most fluid balance calculations assume even fluid balance at ICU admission with the assumption that the anchor weight represents a euvolemic state. This assumption is not supported by evidence and is subject to many potential inaccuracies (over/inadequate fluid resuscitation prior to ICU admission, fluid management on cardiopulmonary bypass, intravenous fluids administered on the general wards, etc.).

In describing the terminology and methodology, the practicality and impact of these definitions in low- and middle-income countries (LMICs) necessitate a separate discussion. In evaluating the potential resources necessary to accurately monitor fluid balance, the “cumulative fluid input and output methodology” may be more resource intensive as it requires a detailed 24-h monitoring. The weight-based methodology has the advantage of being less resource intensive. In LMIC settings, it remains important to use a standardized method to define anchor weight. Weighing neonates and small children is a well-established method in neonatal and pediatric intensive care units (PICU) in LMICs. However, weighing older children and adolescents receiving mechanical ventilation is a challenge as ICU beds capable of weighing patients are less commonly available.

Critically ill neonates warrant separate discussion, as measuring fluid balance and fluid accumulation can be particularly challenging in this population. Special considerations for neonates include increased insensible losses, insensible losses that change with gestational age, and the expected physiologic diuresis over the first 1–2 postnatal weeks. The ascertainment of fluid balance in neonates is further complicated by the fact that sick neonates rarely have Foley catheters in place. Weight-based methods have been clearly shown to be a superior measure of fluid balance in neonates [14, 18, 32–34]. For the neonatal population, the most common anchor weight is the birthweight in the first two postnatal weeks [32]. After this period, there has been little research describing the accurate calculation of an anchor weight.

As the importance of fluid balance has become increasingly clear, an important step is to incorporate fluid balance as a “vital sign” in sick children. As with any other vital sign, fluid balance can be incorporated into clinical rounds and monitoring on a daily basis in sick children. Furthermore, there are high-risk populations where fluid balance may need to be evaluated on a more frequent basis (e.g., CKRT, ECMO). The availability of such data serves as a quality indicator, and quality improvement methodology can be utilized to optimize this [35, 36].

## Areas for research

- Identify the optimal anchor weight needed to calculate fluid balance measurements to be used through a patient’s clinical course (e.g., premorbid weight, dry weight, admission weight, PICU admission weight).
- Systematically study, develop, implement, and evaluate protocols for daily weights in sick children and in different resourced settings (e.g., LMICs).
- Evaluate the optimal method and process to adjust anchor weight for patients hospitalized for extended periods of time.
- Evaluate the optimal method and process to adjust anchor weight for neonates outside of the first 2 postnatal weeks.

## Question 2: What defines fluid overload in sick children?

### Statement: Fluid overload denotes a pathologic state of positive fluid balance associated with a clinically observable event(s), which may vary by age, case-mix, acuity, and phase of illness. No specific threshold of positive fluid balance alone can define fluid overload across all sick children

In order to advance the field, the term *percent cumulative fluid balance* should be utilized to describe the cumulative positive fluid balance over a given time period. The term *fluid overload* should be defined as the degree of positive fluid balance that is associated with adverse patient-centered events such as increased length of mechanical ventilation, length of stay, and/or increased mortality. The threshold and timing of fluid balance that define the pathologic state of fluid overload differ across populations, and disease processes are variable (Table 2 and Supplemental Table S1) [2, 3, 8, 10, 11, 14–16, 18, 20, 23, 27–29, 37–89]. Observational studies have identified important characteristics that impact the thresholds associated with fluid overload, including age, underlying disease process, illness acuity, temporal profile in fluid balance, and available resources. In considering the utilization of thresholds to report fluid balance relative to reporting fluid balance as a continuous variable, one must understand the potential pitfalls to this practice. The utilization of thresholds is subject to potential bias (e.g., influence of extreme values), and this must be appreciated.

**Table 2:** Selected studies evaluating the impact of fluid balance on outcomes

Author, year (*n*)	Study design/population details	Fluid balance (FB) definition	Timing of fluid balance	Outcome	Main findings
Continuous renal replacement therapy					
Goldstein, 2001 (*n* = 21) [8]	-Retrospective, single-center -CKRT, ≤ 18 years old, PICU, 1996–1998	Cumulative fluid input and output	CKRT initiation	Mortality	-FB at CKRT initiation was lower in survivors compared to non-survivors (16.4% ± 13.8% vs. 34.0% ± 21.0%, *p* = 0.03) -FB at CKRT initiation was associated with increased mortality, independent of illness severity score (*p* = 0.03)
Sutherland, 2010 (*n* = 297) [20]	-Prospectively collected multicenter registry data (ppCRRT) -CKRT, < 18 years old, all ICUs 2001–2005	Cumulative fluid input and output	FB at CKRT initiation	Mortality	-FB > 20% associated with increased mortality, independent of illness severity and other clinical factors (aOR 8.5) -Adjusted OR for mortality was 1.03 (95% CI 1.01–1.05), suggesting a 3% increase in mortality for each 1% increase in FB
Neonates					
Schmidt, 2006 (*n* = 999) [29]	-Secondary analysis of the randomized clinical trial of indomethacin prophylaxis in pre-terms (TIPP), multicenter -Extremely low birthweight infants	Weight-based	FB over first postnatal week	Bronchopulmonary dysplasia	-Indomethacin prophylaxis reduced urine volume during the first 4 days of life -The average weight loss after the first postnatal week predicted the development of bronchopulmonary dysplasia
Selewski, 2020 (*n* = 1007) [18]	-Preterm neonates from AWAKEN study -Outcome: MV postnatal day 7	Weight-based	FB over first postnatal week	Mechanical ventilation postnatal day 7	-Multivariable models showed that the following FB variables (1st postnatal week) were independently associated with the need for mechanical ventilation on postnatal day 7: -Peak FB (aOR 1.14, 95% CI 1.10–1.19) -Lowest fluid balance (aOR 1.12, 95% CI 1.07–1.16) -FB on postnatal day 7 (aOR 1.10, 95% CI 1.06–1.13)
Selewski, 2019 (*n* = 645) [14]	-Term neonates from AWAKEN study -Outcome: MV postnatal day 7	Weight-based	FB over first postnatal week	Mechanical ventilation postnatal day 7	-Multivariable models showed that the following FB variables (1st postnatal week) were independently associated with the need for mechanical ventilation on postnatal day 7: -Peak FB (aOR 1.12, 95% CI 1.08–1.17) - Lowest FB (aOR 1.14, 95% CI 1.07–1.22), -FB on postnatal day 7 (aOR 1.12, 95% CI 1.07–1.17) -Negative FB on postnatal day 7 (aOR 0.3, 95% CI 0.16–0.67)
Cardiac surgery					
Hassinger, 2014 (*n* = 98) [11]	-Secondary analysis of a prospective observational study, 2009–2010 -Postoperative cardiac surgery, cardiopulmonary bypass < 18 years of age	Cumulative fluid input and output	End of postoperative day 1	Mortality, ICU length of stay, length of mechanical ventilation	-FB > 5% occurred in 31% of patients at end of postoperative day 1 -FB preceded the development of AKI -FB > 5% associated with length of stay (3.5 days) and were more likely to require prolonged mechanical ventilation (*p* < 0.001
Lex, 2016 (*n* = 1530) [37]	-Prospective cohort 2004–2008 -Calculated FO during first 72 postoperative h	Cumulative fluid input and output	72 h postoperatively	Mortality, prolonged mechanical ventilation (> 72 h)	-89.9% had FB < 5% -Higher FB on the day of the surgery was independently associated with mortality (aOR 1.14; 95% CI, 1.008–1.303; *p* = 0.041) -Cumulative FB on postoperative day 2 was associated with prolonged mechanical ventilation (aOR 1.012, 95% CI 1.005–1.032, *p* = 0.025)
Bailly, 2022 (*n* = 2223) [39] and Neumayr, 2022 (*n* = 2235) [28]	-Observational cohort study of neonates (≤ 30 days) undergoing cardiac surgery -NEonatal and Pediatric Heart Renal Outcomes Network (NEPHRON) study	Cumulative fluid input and output and weight-based	Peak, postoperative day 1, time to negative fluid balance	In-hospital mortality, length of mechanical ventilation, ICU length of stay, hospital length of stay	-Median peak FB 4.9% (IQR 0.4%, 10.5%) as calculated by cumulative fluid balance methodology -Peak FB and postoperative day 1 FB not associated with outcomes -Center variation in obtaining daily weights -Poor correlation between FB calculations and weight-based calculations -Time to first day of negative FB associated with outcomes
General critical care					
Arikan, 2012 (*n* = 80) [3]	-Retrospective, single-center -Mechanically ventilated at 24 h after PICU admission, 2004–2005	Cumulative fluid input and output	Daily FB, peak FB	Oxygenation index, ICU length of stay, mortality	-Peak FB was independently associated with higher peak oxygenation index, (*p* = 0.009) -Peak FB and severe FB (≥ 15%) were both independently associated -Longer duration of ventilation (*p* = 0.008) -Pediatric intensive care unit LOS (*p* = 0.01)
Li, 2016 (*n* = 370) [27]	-Prospective observational study -PICU admissions > 24 h, age 1 month–< 16 years, 2009–2010	Cumulative fluid input and output	FB > 5% at 24 h of ICU admission	Mortality, length of mechanical ventilation, ICU length of stay	-Early FB (≥ 5% at 24 h) developed in 17.3% of patients -Early FB was associated with AKI -Early FB was associated with increased mortality (aOR 1.17, 95% CI 1.01–1.37, *p* = 0.035)
Alobaidi, 2018 (44 studies, *n* = 7507) [23]	-Meta-analysis	Cumulative fluid input and output and weight-based	Not applicable	Mortality, treatment intensity, organ failure, resource utilization	-On adjusted analysis FB was associated with the following: -Increase mortality (aOR 1.06, 95% CI 1.03–1.1, per 1% rise in FO) -Prolonged mechanical ventilation (> 48 h) (aOR 2.14, 95% CI 1.25–3.66) -AKI (aOR 2.36, 95% CI 1.27–4.38)
Alobaidi, 2020 (*n* = 1017) [2]	-Retrospective cohort study -Provincial PICU dataset (Alberta, Canada) -All admission 2015	Cumulative fluid input and output	Daily cumulative FB, peak FB during first 10 ICU days, duration of fluid accumulation	ICU mortality, Major Adverse Kidney Events within 30 days (MAKE_30_), ICU length of stay, length of mechanical ventilation	-Proportion of patients with peak FB > 10% was 32.7% (29.8–35.7%) and > 20% was 9.1% (7.4–11.1%) -Peak FB was associated with greater PICU mortality (OR 1.05; 95% CI 1.02–1.09; *p* = 0.001) -Greater peak FB was associated with the following: -Major Adverse Kidney Events within 30 days (OR 1.05; 95% CI 1.02–1.08; *p* = 0.001) -Length of mechanical ventilation (B coefficient 0.66; 95% CI 0.54–0.77; *p* < 0.001) -Length of PICU stay (B coefficient 0.52; 95% CI 0.46–0.58; *p* < 0.001) -The rate of fluid accumulation was associated with adverse outcomes
ECMO					
Selewski, 2012 (*n* = 53) [16]	-Retrospective, single-center -CKRT, all patients treated with CKRT and ECMO, 2006–2010	Weight-based	FB at CKRT initiation, FB at CKRT discontinuation	ICU mortality	-Median FB at CKRT initiation was lower in survivors (24.5% vs. 38%, *p* = 0.006) -Median FB at CKRT discontinuation was lower in survivors (7.1% vs. 17.5%, *p* = 0.035) -Models evaluating fluid removal consistently showed that the degree of FB at CKRT initiation consistently predicted mortality
Selewski, 2017 (6 centers, *n* = 756) [15]	-Retrospective chart review of all children < 18 years treated with ECMO, 2006–2010	Cumulative fluid input and output	FB at ECMO initiation, peak FB on ECMO, FB at ECMO discontinuation, change in FB on ECMO	In-hospital mortality, ECMO mortality, duration of ECMO	-Median peak FO on ECMO was 30.9% (IQR, 15.4, 54.8) -Peak FB during ECMO: 84.8% ≥ 10%; 67.2% ≥ 20% and 29% ≥ 50% -Multivariable analysis showed that multiple measures of FB were associated with outcomes: -Peak FB on ECMO (aOR 1.09, 95% CI 1.04–1.15) predicted mortality on ECMO -FB at ECMO initiation (aOR 1.13, 95% CI 1.05–1.22) and peak fluid overload (aOR 1.18, 95% CI 1.12–1.24) both predicted hospital morality
Gorga, 2020 (6 centers, *n* = 357) [10]	-Retrospective chart review of all children < 18 years of age concurrently treated with ECMO and CKRT six tertiary care children’s hospitals	Cumulative fluid input and output	FB at CKRT initiation, FB at CKRT discontinuation, change in FB on ECMO	In-hospital mortality, ECMO mortality, duration of ECMO	-Median FB at CKRT initiation was 20.1% (IQR 5, 40) and was significantly lower in ECMO survivors vs. non-survivors (15.3% vs. 30.5% *p* = 0.005) and in hospital survivors vs. non-survivors (13.5% vs. 25.9%, *p* = 0.004) -Multivariable analysis, FB at CKRT initiation (aOR 1.09, 95% CI 1.00–1.18, *p* = 0.045) and at CKRT discontinuation (aOR 1.11, 95% CI 1.03–1.19, *p* = 0.01) were independently associated with hospital mortality
Low-middle income countries					
Rusmawatiningtyas, 2021 (*n* = 665, Indonesia) [39]	-Retrospective cohort, single-center from 2014 to 2019 -Sepsis patients admitted to PICU	Cumulative fluid input and output	Peak FB	Mortality	-29% had a FB > 10% -Higher mortality associated with FO > 10% (HR 9.6, 95% CI 7.4–12.6)
Marquez-Gonzalez, 2019 (*n* = 242, Mexico) [40]	-Prospective cohort, single-center -Septic shock in children 1–17 years from 2011 to 2016	Cumulative fluid input and output	Peak FB	Mortality	-FB ≥ 10.1% at 24 h (29.8%), 48 h (23.1%), 72 h (28.1%), 96 h (32.2%) -%FB ≥ 10.1% at 96 h was related to a higher mortality at 28 days on adjusted analysis for hemodynamic profile model (aHR 2.6, 95% CI 1.9–5.6), refractory septic shock model (aHR 2.5, 95% CI 1.6–5.6), malnutrition model (aHR 8.3, 95% CI 3.5–14)
Maitland, 2011 (*n* = 3141, Uganda, Kenya, Tanzania) [41]	-RCT-3 arms: (1) 5% albumin bolus (2) normal saline bolus (3) no bolus -Inclusion: children 60 days to 12 years with severe febrile illness	Not applicable	Not applicable	48-h mortality, increased intracranial pressure, pulmonary edema, mortality and/or neurologic sequelae at 4 weeks	-Clinical signs of *fluid overload* (defined as pulmonary edema or increased intracranial pressure) occurred in only 2% of children regardless of fluid bolus or not -RR of mortality at 48 h was 1.45 (1.13–1.86, *p* = 0.003) for any bolus vs. no bolus

**Fig. 1:**
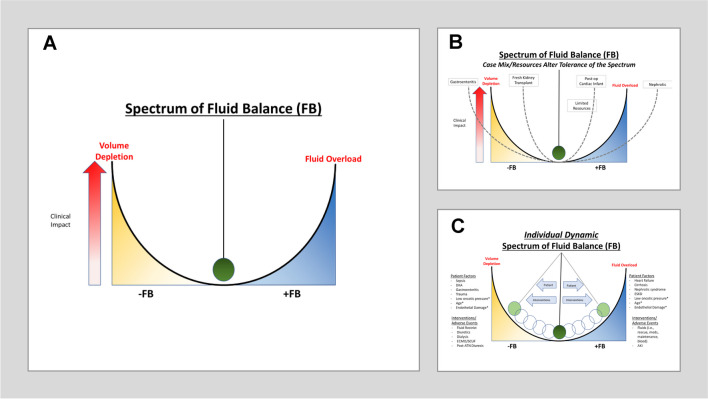
The Spectrum of Fluid Balance [90]. There is a spectrum of fluid balance for the sick child that is a U-shaped curve. (A) This spectrum can swing between positive fluid balance (+FB) and negative fluid balance (-FB) with varying levels of clinical impact depending on the severity of the abnormal fluid balance. (B) Depending on the case-mix and/or resources available, the U-shaped curve may have more or less tolerance for a positive or negative fluid balance. These depict examples of different scenarios where a patient population may have greater or lesser tolerance of a +FB or -FB scenario. (C) For any given sick child, there are several factors (i.e., host factors, interventions, adverse outcomes) that may push them towards a greater +FB or -FB or pull them back towards a state of neutral fluid balance at the center. These may vary over the course of the hospitalization and can be constantly changing

With the paradigm shift on how to ideally define fluid balance, there must be an associated shift in how we describe its epidemiology. In defining fluid overload as positive fluid balance associated with adverse clinical events, it is implicit that not all states of positive fluid balance are in fact deleterious. Furthermore, the full spectrum of fluid balance should be considered, including negative fluid balance. It is reasonable to think that negative fluid balance is not always beneficial for the patient, and that in certain disease states, may become detrimental to the patient. This paradigm and its associations with various disease states and clinical factors are illustrated in Fig. 1.

A major gap and limitation in the epidemiology of fluid balance is understanding the full continuum of fluid balance, particularly iatrogenic and clinically important negative fluid balance or clinical dehydration. The incidence of positive fluid balance, including temporal profile and magnitude, varies by center, definition, reported thresholds, and predetermined thresholds set as “clinically meaningful fluid overload” (Supplemental Table S1). The standardization of definitions and reporting will improve the understanding of the full spectrum of fluid balance and allow us to better define and describe fluid overload. The literature to date shows wide ranges in the incidence of fluid overload among critically ill children depending on the threshold used: 17–70% for > 5%, 4.5–56% for > 10%, 9–33% for > 20% (Supplemental Table S1).

In recent years, pediatric fluid research shifted from identifying absolute thresholds of fluid overload to understanding the impact of timing and trajectory of fluid accumulation on outcomes. In a single center, retrospective analysis of post-cardiac surgery patients, the median peak positive fluid balance was 4.5% and most commonly occurred on postoperative day 2 [91]. Among a cohort of mechanically ventilated children across five PICUs, the mean daily cumulative positive fluid balance was positive 20–35 ml/kg on hospital days 1–3 [92]. An evaluation of over 1000 critically ill children in Canada found that the positive fluid balance increased with each subsequent ICU day from 1.6% on hospital day 1 to 16.4% on hospital day 10 [2]. This data taken together emphasizes the importance of considering timing, magnitude, and trajectory in evaluating the impact of fluid balance.

A significant amount of work has gone into understanding the case mix and factors associated with derangements in fluid balance and the development of fluid overload (Supplemental Table S1). In post-cardiac surgery populations, young age, severity of AKI, longer cardiopulmonary bypass time, and surgical complexity were associated with derangements in fluid balance [11, 13, 37, 93]. In general, PICU populations, younger age, AKI, inotrope use, ventilatory support, shock diagnosis, and admission to a medical/surgical unit vs. a cardiac unit were associated with higher fluid balance categories [2, 3, 27, 94, 95]. Given the associations between positive fluid balance and adverse outcomes, including mortality, further investigation for modifiable factors as targets to enrich clinical trials and be actionable for intervention are needed.

Another important subset of patients with little data is in LMICs, like South Africa, that has reported lower proportions of critically ill children with fluid overload (3% with > 10%). The same cohort also had similar median peak positive fluid balance of 3.5% for their study period as compared to high-income settings [96]. Further work is needed to better understand whether different thresholds are associated with adverse events attributed to a positive fluid balance in often less resources settings encountered in LMICs.

### Triggers and interventions

**Fig. 2:**
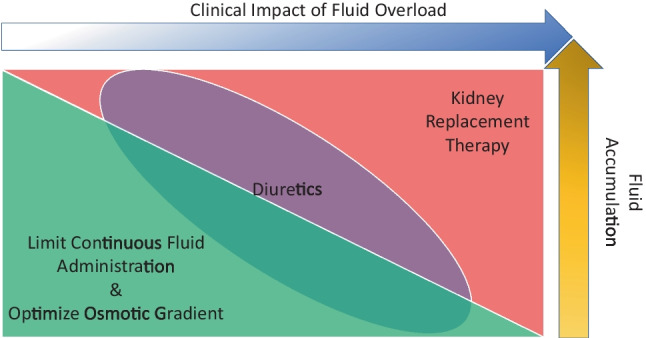
Overlapping Interventions to Manage Fluid Balance. Interventions to manage fluid balance are overlapping based on the degree of fluid accumulation and clinical impact of the fluid. These interventions include limitation of continuous fluids, targeted diuretic therapy, and then consideration for the use of kidney support therapy. Green triangle represents limitations to continuous fluids and optimizing the osmotic gradient. Red triangle represents the need for renal replacement therapy. Purple oval outlines the role of diuretics while patients during both limitation of fluids and need for renal replacement therapy

Two types of interventions can be implemented to modify fluid accumulation: limiting fluid administration and facilitating active fluid removal through pharmacologic (i.e., diuretics) and/or mechanical (i.e., kidney replacement therapy (KRT)) approaches. In sick children, administered fluids have varying degrees of necessity and include those that are life sustaining (i.e., resuscitation, blood products, medication, nutrition) and those that may be restricted (i.e., intravenous fluids, carriers for medications). A large proportion of fluid administered to critically ill children comes in forms that may be restricted, particularly maintenance fluids [97, 98]. We suggest clinicians and researchers study fluid balance in an overlapping but sequential manner: limitation, targeted diuretic therapy, and then consideration for the use of mechanical fluid removal (i.e., KRT). Figure 2 demonstrates a theoretical framework of the potential overlapping interventions based on the degree of fluid accumulation and its clinical impact.

Another important clinical consideration is to begin to consider preventative measures or fluid stewardship programs aimed at mitigating fluid accumulation in high risk populations prior to the development of overt fluid overload [3, 97, 99]. This could be done by identifying patients at high risk for developing fluid overload and/or kidney dysfunction (i.e., predictive enrichment and clinical risk prediction) that would further magnify the accumulation of IVF and by taking a targeted approach to more aggressively limit a positive fluid balance. This may include the deployment of several tools (e.g., renal angina index, novel biomarkers, furosemide stress test, fluid overload, and kidney injury score (FOKIS)) in high-risk populations [99–103]. These tools can be utilized to identify high-risk patients who would most benefit from a precision approach to fluid management with the targeted use of diuretics and kidney support therapy. A clinical trial currently underway that is utilizing this approach is the “Trial in AKI using NGAL and Fluid Overload to optimize CRRT Use” (TAKING FOCUS 2). This trial is evaluating the impact of sequentially mobilizing urinary biomarkers and the furosemide stress test to identify patients at risk for the development of fluid overload and severe AKI [104, 105].

## Areas for future research

- Further understanding of the incidence of both positive and negative fluid balance in all sick children (acute, critically ill, neonates, post-cardiac surgery, oncologic patients, etc.).
- Further delineate the thresholds of positive fluid balance whereby the development of fluid overload is most likely to occur across all sick children.
- Understand the impact of timing and trajectory of positive fluid balance on outcomes.
- Identify the risk factors and potential modifiable factors for positive fluid balance and fluid overload in all sick children.
- Further study and understanding of the interplay and impact of intravascular volume status and total body volume. Further work is needed to define and standardize the terminology including but not limited to hypervolemia, euvolemia, and hypovolemia relative to total body volume and intravascular volume.
- Develop objective and reproducible measures of edema.
- Identify the potential associations of negative fluid balance and clinical dehydration on clinical outcomes (such as venous thromboses).
- Identify the incidence, risk factors, and associated outcomes of negative and positive fluid balance in children in LMICs. This is urgently needed to better understand and put into clinical context the findings of the FEAST trial.
- Identify thresholds for intervention in various case mixes of sick children.
- Evaluate the effectiveness of different interventions on the degree of positive fluid balance and the impact on fluid overload and associated outcomes.
- Evaluate the effectiveness of a preventative strategy or fluid stewardship interventions. (i.e., reduce fluid administration, improve cumulative fluid balance, improve physiological outcomes, and improve patient reported outcomes).

## Question 3: What are the challenges to translating observational data to clinical management of fluid balance?

### Statement: The literature describing the association of fluid balance on outcomes is based on observational studies. Evidence from observational data alone cannot establish a causal relationship between fluid balance and outcomes

In order to move the field forward and improve outcomes related to fluid balance, a better understanding and establishment of the causal link between fluid balance and outcomes are needed. The lack of randomized trials in hospitalized children and complexity of evaluating fluid balance in critically ill patients make it challenging to causally link fluid balance to outcomes. In order to have the highest likelihood of success, these trials would benefit from utilizing many of the previously described tools to identify the highest risk patient populations that would potentially benefit. We provide further suggestions on optimizing study design and outcomes to inform the development of trials focused on the impact of fluid balance on outcomes.

### Study designs

**Fig. 3:**
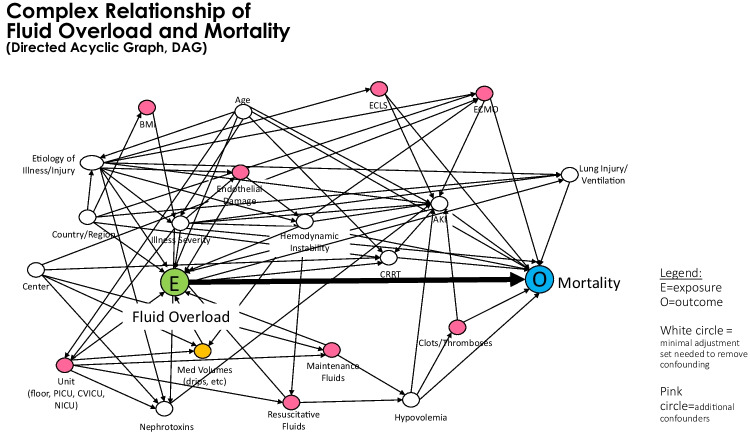
Directed Acyclic Graph of Fluid Overload and Mortality [90]. The directed acyclic graph (DAG) above is an example of the utilization of this tool to describe the complex relationship between fluid overload and mortality in sick children. Different DAGs would be necessary to evaluate positive or negative fluid balance or other fluid states and different outcomes. The variables depicted in this figure are likely not exhaustive and additional nuanced variables could be considered (e.g., genetics). The figure is meant to be as comprehensive as possible with current knowledge-to-date to demonstrate the complexity of the relationship and the importance that all factors are considered to minimize bias when trying to use observational studies to evaluate the fluid overload-mortality relationship and its potential for a causal pathway. Arrows depict a known associated risk relationship (whether that is a positive or negative relationship) E=the primary exposure of interest (fluid overload in this example) O=the primary outcome of interest (mortality in this example) White circles = represent variables that are confounders in the primary exposure-outcome relationship and would be the required minimal adjustment variables to account for in order to get a fully adjusted analysis of the primary exposure-outcome relationship (i.e., the minimal adjustment set) Pink circles = represent additional variables that are also confounders but not necessary for a minimal adjustment set evaluating the primary exposure-outcome relationship Orange circle = ancestor to primary exposure of interest

Table 3 outlines some of the ongoing and seminal studies that have been done to date in adults and children evaluating the impact of fluid balance on clinical outcomes. To date, most data surrounding the impact of fluid balance on outcomes are derived from observational studies. Observational data are important tools that allow us to study new concepts and exposures that are difficult to randomize. The confounders that have an association with both the exposure (i.e., fluid balance) and outcomes must be realized and controlled for. A useful tool for researchers is a directed acyclic graph (DAG). A DAG is a diagrammatic tool to allow researchers to visualize confounders, mediators, and intermediaries on pathways, as well as other relevant exposures. A DAG developed from observational studies describing the interactions of fluid overload and morbidity and mortality is presented in Fig. 3. A detailed understanding of the complex relationship between patient characteristics, fluid balance, and outcomes from observational studies will serve to inform clinical trial design.

**Table 3:** Planned, ongoing, or recent randomized clinical trials in adult/pediatric populations focused on fluid accumulation in critical illness (with or without AKI)

Trial	Design	Size	Eligibility	Outcome	Intervention
Efficacy, safety, pharmacokinetics, and pharmacodynamics study of tolvaptan in pediatric congestive heart failure (CHF) patients with volume overload (NCT03255226)	Interventional, open-label	60	-Pediatric patients with congestive heart failure -Patients with “volume overload” despite having received diuretic therapies	-Ratio of subjects for whom the body weight is decreased from baseline (time frame: day 1, day 4) -Ratio of subjects for whom the body weight is decreased by 1.7% or more from the baseline -Change in the amount of daily urine volume from baseline (time frame: day 1 to day 9)	-Tolvaptan 1% granules or tolvaptan 15 mg tablet daily
Phase III study of furosemide continuous infusion versus ethacrynic acid continuous infusion in children undergoing cardiac surgery: randomized double blind controlled clinical trial [106]	Interventional, randomized, quadruple masking (participant, care provider, investigator, outcomes assessor)	74	-Children with congenital heart disease undergoing cardiac surgery -Intraoperative aortic cross clamp > 90 min or interventional catheterization procedures with postoperative inotrope score over 20 -Signs of fluid retention after surgical procedures	-Primary outcome measures: mean total urine output production in the first postoperative day -Secondary outcome measures: mean creatinine and NGAL values	Drug: furosemide (intravenous, continuous infusion, 0.2–0.8 mg/kg/h for 24 h Drug: ethacrynic acid (intravenous, continuous infusion, 0.2–0.8 mg/kg/h for 24 h
Ultrafiltration Therapy Registry Using Aquadex (ULTRA-Peds) ULTRA-Peds: a multicenter data registry for outcomes for pediatric volume overload (NCT04644731)	Observational (cohort, registry)	500	Age < 21 years or younger Patient who is to undergo or underwent Aquadex therapy for “fluid overload” as local standard of care	-Multiple outcomes: treatment survival, survival at ICU discharge, change in kidney function, hemodynamic stability at initiation of UF therapy, hemodynamic stability during treatment course, need for vasoactive medication, change “% fluid overload” during treatment course, ICU LOS, change in PRISM III score, Aquadex-related adverse events	
Trial in AKI using NGAL and Fluid Overload to optimize CRRT Use, TAKING FOCUS 2 (NCT03541785) [104, 105]	Observational study	1427, ongoing	Age 3 months– < 25 years	Primary outcome measures: clinical decision support success (CDS). Secondary outcome measures: furosemide stress test (FST) standardization	
Prospective, multi-center, single-arm, observational study. US FDA 522 pediatric post market surveillance study (NCT04608149)	Observational (patient registry)	35	Inclusion: informed consent, subject weight 2.5–10 kg, admitted to ICU, parental or LAR consent to receive full supportive care through aggressive management utilizing all available therapies for > 96 h, subject has a clinical diagnosis of acute kidney injury	Survival at CKRT discontinuation, evaluation subject survival at CKRT discontinuation, survival at intensive care unit (ICU) discharge	Continuous kidney replacement therapy with Carpediem system
Less is more? - a feasibility study of fluid strategy in critically ill children with acute respiratory tract infection (NCT02989051) [107]	Interventional, randomized controlled, open-label, feasibility	34	Inclusion criteria: admitted to the PICU, intubated and mechanically ventilated (anticipated duration ≥ 72 h, acute infectious lung disease	Primary outcomes: cumulative fluid balance, body weight. Secondary outcomes: duration of mechanical ventilation, oxygenation indices, mortality	Compared “conservative” fluid management strategy (maximal daily fluid intake of 70% of normal requirements) to “liberal” fluid management strategy (fluid regimen of > 85% of normal fluid recommendations) in mechanically ventilated patients
Restricting volumes of resuscitation fluid in adults with septic shock after initial management: the CLASSIC randomised, parallel-group, multicentre feasibility trial [108]	Multicenter RCT (pilot feasibility)	151	Adult; in ICU; fulfilled sepsis criteria in prior 24 h; suspected or confirmed circulatory impairment; received 30 mL/kg fluid in prior 6 h; shock defined by ongoing NE infusion	Co-primary outcomes: (1) amount of resuscitation fluid (cumulative fluid given for circulatory impairment in the first 5 days); (2) amount of resuscitation fluid given after randomization during entire ICU stay Secondary: total fluid input in ICU at day 5 and entire ICU stay; fluid balance at day 5 and entire ICU stay; no. of PV; serious AE Exploratory: death 90 days; VFD; KRT-free days; no. ischemic events; rates of AKI (KDIGO); max SCr change	Protocolized resuscitation—randomized after initial resuscitation. No colloids. Restrictive: crystalloid boluses 250–500 mL allowed for evidence of “severe hypoperfusion defined by one of the following: (1) lactate > 4; (2) MAP < 50; (3) mottled kneecaps; (4) oliguria (first 2 h only); response evaluated after 30 min Standard: crystalloid boluses given to improve hemodynamic profile as directed by clinicians; response evaluated after 30 min
Restrictive fluid management versus usual care in acute kidney injury (REVERSE-AKI) [109]	Multicenter RCT (pilot feasibility)	100	Adult; in ICU (> 12 h to < 72 h); arterial line; had AKI not receiving KRT; judged by treating clinical to NOT be hypovolemic; likely to remain in ICU > 48 h	Primary endpoint: cumulative FB 72 h after randomization. FB calculated as total fluid output (urine, drain losses, GI losses, UF by KRT) minus total fluid input (IV + oral). Insensible not considered Secondary: duration of AKI (days) by KDIGO, no. of patients receiving KRT, cumulative FB at 24 h after randomization and at ICU discharge, cumulative dose diuretics Exploratory: VFD at 14 days, ICU-free days at 14 days, KRT-free days at 14 days and 90 days, no. of patients with AE, no. PV, feasibility measures	Restrictive fluid management: bundled recommendations for 7 days from randomization or ICU discharge including: restricting total daily fluid input to only medications + nutritional fluids + blood products, use of maintenance IV fluid only permitted if EN not tolerated and PN contraindicated, fluid bolus therapy only given as “clinically deemed necessary,” matching fluid output (unrestricted diuretic use) to fluid input whenever possible to achieve negative cumulative fluid balance but always < 300 mL/day, if FB target is not achieved—consider KRT to remove required fluid (commencing KRT not mandated) Usual care: fluid management at discretion of treating clinical team
Restrictive versus Liberal Fluid Therapy in Major Abdominal Surgery (RELIEF) trial [110]	Multicenter RCT	3000	Adult; increased risk for complications after major abdominal surgery; operative duration > 2 h; expected hospital stay 3 days. RISK criteria included the following: age > 70 years; presence of heart disease; DM; kidney impairment or morbid obesity. Excluded for urgent/emergent, liver resection, laparoscopic surgery or kidney failure	Primary outcome: disability-free survival at 1 year; disability defined as a persistent impairment in health status (lasting ≥ 6 months) (WHODAS questionnaire) Secondary: rates of AKI (KDIGO), composite of mortality or major septic complications (sepsis, SSI, anastomotic leak, pneumonia) at 30 days; lactate at 6 h; peak CRP; rates of blood transfusion; ICU and hospital stay; rates of KRT at 90 days	Liberal: design to reflect usual practice; bolus 10 mL/kg given at induction; 8 mL/kg/h until end of operation; postoperative 1.5 mL/h/h for 24 h but could be increased or decreased in response to complications of overload or hypovolemia Restrictive: aimed for net zero FB. Less than 5 mL/kg at induction; no other IV fluids before surgery; 5 mL/kg/h until end of operation; postoperative 0.8 mL/kg/h—adjusted for 24 h. Oliguria was not used as an indication for additional administration of IV fluids
Fluids and Catheters Treatment Trial (FACTT) [111]	Multicenter RCT	1000	Adult; intubated; receiving PPV; P/F ratio < 300 and bilateral infiltrates on CXR. Excluded for: ALI > 48 h; chronic conditions that would independently modify survival or compliance with protocol; estimated 6-month mortality > 50% (i.e., advanced cancer)	Primary outcome: mortality at 60 days prior to discharge home Secondary: VFD at 28 days; ICU-free days at 28 days; organ failure-free days at 28 days; KRT through day 60	All patients received IMV as per ARDS network protocol of lower TV Conservative: insertion of CVC with CVP and/or PAOP. Hemodynamic management started within 2 h of randomization; monitored every 4 h; and continued for 7 days or until 12 h after able to breath spontaneously Liberal: usual care; within spectrum of protocol
SQUEEZE (NCT03080038) [112]	Multicenter RCT	400*	Age 29 days to 18 years; persistent signs of shock including one or more of the following: (1) vasoactive dependence, (2) hypotension, and (3) abnormal perfusion; suspected or confirmed septic shock; received initial fluid resuscitation: minimum of 40 mL/kg as fluid boluses in prior 6 h or 2 L (if ≥ 50 kg); fluid refractory septic shock define as all persistent signs	Primary outcome: difference in time to shock reversal (in hours; within 14 days) Secondary: differences in PELOD-2 score at 28 days; rates of AKI at 28 days; VFD at 28 days; rates of complications of fluid overload at 14 days (soft tissue edema; pulmonary edema; pleural effusion requiring drainage; ACS; diuretic exposure); rates of complications of inotrope/vasopressors (digital ischemia; amputation revision; compromised bowel perfusion); PICU and hospital duration of stay; mortality (28, 90 and hospital); PICU readmission at 28 days	Fluid sparing resuscitation strategy: two tiers Tier 1: initiate IV/IO vasoactives immediately. Further fluid bolus therapy should be avoided; small volume isotonic fluid boluses may be required due to the following: (1) clinically unacceptable delay in ability to initiate vasoactives or (2) documented intravascular hypovolemia Tier 2: vasoactives should be preferentially titrated/escalated to achieve recommended ACCM hemodynamic goals. Further fluid bolus therapy should be avoided; small volume isotonic fluid boluses may be provided if required due to documented intravascular hypovolemia Usual care resuscitation strategy: decisions regarding fluid bolus therapy and/or initiation or escalation of vasoactive therapy are at the discretion of the treating clinical team. Suggest that vasoactives not be started until > 60 mL/kg isotonic fluids are given (> 3 L for children ≥ 50 kg). Advised to follow ACCM CPG for resuscitation

Abbreviations: *RCT* randomized controlled trial, *NE* norepinephrine, *PV* protocol violations, *AE* adverse events, *SCr* serum creatinine, *FB* fluid balance, *EN* enteral nutrition, *PN* parenteral nutrition, *ACS* abdominal compartment syndrome, *SSI* surgical site infection

^*^Planned recruitment target

In designing future observational studies, we suggest researchers to incorporate novel approaches to analysis to strengthen these studies. The first is to utilize appropriate sample sizes and to switch from logistic regression modelling (i.e., odds ratios) to linear or log-linear modelling (i.e., risk differences and risk ratios) when feasible. This analytic strategy will improve our understanding to look at risk ratios, risk differences, or attributable risks to determine the degree that a particular risk may be contributing to outcomes of interest.

Traditional randomized controlled trials, or explanatory trials, have been considered the gold standard to delineate causality in between an intervention and outcome. Randomized controlled trials can be challenging to design and implement, particularly for relationships as complex as outcomes associated with fluid balance. In recent years, pragmatic trials have been increasingly utilized to study approaches to management fluid, with fluid balance representing a key process and outcome measure. Pragmatic trials are characterized by simple eligibility, broad inclusion, simple data collection and use of core outcome sets, easily applied intervention, and group randomization (e.g., ward level, unit level, hospital level) [113]. Each of these characteristics aims to optimize the ease of the trial implementation, to evaluate interventions in “real world” scenarios, and to enable ease of translation of findings into practice. Several pragmatic and novel clinical trial design approaches have recently been applied (i.e., stepped-wedge implementation; cluster cross-over, quasi-experimental) in a range of clinical contexts that may be well-suited to evaluation of complex interventions for fluid management in sick children. Adaptive trial designs represent another important tool that may be utilized to study fluid balance in sick children. Simply put, such studies allow for modifications of study design that may be implemented at predefined interim analysis [114].

Given the complexity of fluid balance in sick children and our limited understanding of whether or not a direct causal pathway exists with adverse outcomes (i.e., mortality), we suggest innovative trial design be incorporated into future studies. A good example is the FLUID trial which proposes a hospital-wide (adults and children) cluster-randomized crossover trial to evaluate outcomes between 0.9% NaCl and Ringer’s lactate fluids (https://clinicaltrials.gov/ct2/show/NCT02721485, NCT02721485) [115].

### Outcomes

The association of fluid overload and short-term outcomes during the initial hospitalization has been the most frequently reported study design, with mortality being the most common outcome. While mortality remains important, it is relatively uncommon, necessitating the need for alternative outcomes (Table 4). These outcomes include circumstances where fluid accumulation has led to an escalation in care due to the development of deteriorating clinical status or worsening organ function, including increased receipt of respiratory support, increased oxygenation index, increased ionotropic support, worsened cardiac dysfunction, subsequent or worsening AKI, receipt of KRT, or receipt of extracorporeal life support (ECLS). By extension, there are studies that report on outcomes related to the duration of such interventions (e.g., mechanical ventilation, ECLS, KRT) or hospitalization (ICU or total hospital length of stay) based on degrees of positive fluid balance. Additionally, ICU mortality and in-hospital mortality are commonly reported as outcomes of interest in observational studies. To date, there are few data describing the cost attributable to abnormalities in fluid balance in children.

**Table 4:** Summary of the spectrum of endpoints in trials focused on fluid accumulation/overload

Categories of trial endpoints	Description
Process of care/physiological	
Fluid balance	Differences in FB—daily and cumulative FB Differences in FB targets—daily and cumulatively
Weight	Differences in weight—daily and cumulative weight gain/loss
Complications of fluid accumulation	Defined as the presence of soft tissue edema, pulmonary edema, pleural effusion requiring drainage, and abdominal compartment syndrome requiring intervention
Acute kidney injury	Defined by KDIGO. This could be further classified as event (yes/no), severity, progression, and duration/persistence
Inflammation	Defined by the blood/tissue mediators (e.g., WBC, CRP, cytokines [IL-6]) as well as mediators of vascular integrity and endothelial function (e.g., VEGF)
Organ dysfunction	Defined by composite organ scores (e.g., PELOD, PODIUM)
Clinical	
Sepsis	Defined by SEPSIS-3 criteria
Chronic kidney disease	Defined by validated and guideline supported SCr-based criteria as well as other measures of kidney damage (e.g., hypertension, proteinuria) and kidney failure requiring either KRT or transplantation
Mortality	Defined by status at a given time. This can also be assessed in various composite endpoints (e.g., persistent organ dysfunction, MAKE, event-free survival (ventilator, KRT, ICU, disability))
Alive and at home	Defined as a composite of survival and days out-of-hospital and at home. This could be looked at various time points. This would integrate re-hospitalization
Health-related QoL	Differences in HRQoL. A recent review highlighted the most commonly utilized instruments including the PedsQL™, the Health Utility Index, the Child Health Questionnaire, and the 36-Item Short Form Survey to evaluate the impact of critical illness on HRQoL [116, 117]
Cognitive development	Defined by validated measures of cognitive (neuropsychiatric) development according to age/sex-specific milestones. This could include any impairment (yes/no) or gradient-specific impairment
Physiological development	Defined by validated measures of physical development according to age/sex-specific milestones
Disability	Defined by the presence of any disability (across a range of domains using a validated pediatric instrument) as well as severity and duration. A multitude of scales exist including but not limited to the Functional Status Scale, the Pediatric Overall Performance Category, and Pediatric Cerebral Performance Category Scales [118–121]
Health services use	
KRT	Defined by commencement of KRT as well as duration
IMV	Defined by commencement of IMV as well as duration (and other events—like tracheostomy). This could also likewise incorporate non-invasive ventilation
Vasoactive support	Defined by commencement of any inotropic or vasopressor support as well as duration
ECLS	Defined by commencement of ECLS (VV-ECMO or VA-ECMO) as well as duration
ICU	Duration of ICU stay and/or readmission to ICU
Hospital stay	Defined by duration of hospital stay as well as pre- and post-ICU stay

Recent work suggests that AKI and disorders of fluid accumulation may impact the long-term outcomes associated with other organs and multisystem disorders. Recent work by the Life After Pediatric Sepsis Evaluation (LAPSE) investigators has evaluated the association of severe AKI with the development of new comorbidities in children following critical care admission for sepsis. In this multicenter study, children with severe AKI had an increased odds of death or new substantive functional morbidity (adjusted odds ratio, 2.78; 95% CI, 1.63–4.81; *p* < 0.001) [122]. More recently, the functional outcomes of children treated with CKRT were assessed by the Functional Status Scale (FSS) in a single-center retrospective study of 45 children. In this study, 31 (69%) had worse FSS score at PICU discharge, and 51% of the children had new morbidity (defined by the FSS score). Furthermore, on adjusted analysis, the degree of fluid overload at CKRT initiation predicted a worse FSS score in this population [118]. In critically ill adults, the degree of positive fluid balance at ICU discharge predicted impaired mobility and discharge to another healthcare facility [123]. To date, there has not been a systematic evaluation of the impact of fluid balance on long-term outcomes in sick children. Future studies should include a multidisciplinary evaluation of outcomes including respiratory, cardiovascular, neurodevelopmental, health-related quality of life, and functional outcomes.

In recent years, the Major Adverse Kidney Events (MAKE) have been proposed to be included as a composite outcome to be utilized and reported in all effectiveness clinical trials in AKI. This composite outcome includes death, new kidney support therapy, or persistent serum creatinine greater than two times baseline. This outcome can be assessed at 30, 60, and 90 days. An important area for future research is to understand the applicability of MAKE outcomes to children and to revise it as necessary to fit pediatric and neonatal outcomes.

## Areas for future research

- Pragmatic randomized trials such as crossovers, cluster RCTs, stepped-wedge, or other quasi-experimental designs are likely needed to further understand the complex relationship of fluid balance and mortality, and whether or not a causal link exists.
- Capitalize on existing networks such as NINJA, collaborative pediatric critical care research network to conduct multicenter randomized trials to answer the missing epidemiological questions surrounding fluid balance and mortality and other clinical outcomes.
- Effects on outcomes of “isolated positive fluid balance” in patients with “normal” kidney function.
- Prospective association of fluid overload and organ function (causally or temporally associated).
- Verification if fluid overload is “continuously” associated with outcomes (i.e., FO 10% leads to an ICU stay of 7 days; 15%, 8 days; 20%, 10 days) or if there is a “dichotomic” threshold (i.e., FO above/below the clinically relevant threshold has significant outcomes implications).
- Improved understanding of the etiology of fluid balance and the development of the state of fluid overload. This includes contributions from severity of illness, disease pathophysiology, timing, impaired fluid clearance (e.g., AKI), inadequate fluid stewardship, endothelial dysfunction, inflammation, and capillary leak.

## Conclusion

The understanding of how fluid balance impacts the clinical care and outcomes of sick children is evolving. This includes a recognition that observational data alone does not determine causality. Fluid balance is an objective measure that ideally will be considered daily in patient care. Fluid overload is a pathologic state associated with positive fluid balance and clinically observable events. Armed with these important distinctions, further research will be able to better discern the relationship between fluid balance and fluid overload as well as help evaluate future clinical practices. 

### Supplementary Information

Below is the link to the electronic supplementary material.

Supplementary file1 (DOCX 153 KB)
