# Supplementary material for: Fluid assessment, fluid balance, and fluid overload in sick children: a report from the Pediatric Acute Disease Quality Initiative (ADQI) conference
Source: Pediatr Nephrol. 2023 Nov 7;39(3):955–79. doi: 10.1007/s00467-023-06156-w (PMC10817849; doi:10.1007/s00467-023-06156-w)
Supplement: Supplementary file 1 — Supplementary file1 (DOCX 153 KB) [file 467_2023_6156_MOESM1_ESM.docx]

| Supplemental Table 1: Studies Evaluating the Impact of Fluid Balance on Outcomes | | | |
| --- | --- | --- | --- |
| Author, Year (N) | Study design/ population details | Fluid Balance (FB) Definition | Main Findings |
| **Continuous Renal Replacement Therapy** | | | |
| Goldstein, 2001 (n=21) [8] | - Retrospective, single-center  - CRRT, ≤18 years old, PICU, 1996-1998 | Cumulative fluid input & output | - FB at CRRT initiation was lower in survivors compared to non-survivors (16.4% ± 13.8% vs 34.0% ± 21.0%, p =0.03)  - FB at CKRT initiation was associated with increased mortality, independent of illness severity score (p=0.03). |
| Gillespie, 2004  (n=77) [42] | - Retrospective, single-center  - CRRT for AKI or FO, ≤20 years old, 1993-2002 | Cumulative fluid input & output | - FO >10% at CRRT initiation were at 3.02 times greater risk of mortality [95% CI 1.50-6.10, P=0.002]    - High-dose convective clearance did not portend improved mortality |
| Foland, 2004, 2004 (n=113) [7] | - Retrospective, single center  - CRRT, PICU, 1997-2003 | Cumulative fluid input & output | - Median FB was significantly lower in survivors vs. non-survivors for:  -- All patients (7.8% vs. 15.1%, p =.02]  -- Patients ≥ 3-organ MODS (9.2% vs. 15.5%, p =.01)  - %FO (per 10% increase) was independently associated with survival in patients with ≥ 3-organ MODS (aOR 1.78, 95%CI, 1.13, 2.82 p =.01) |
| Michael, 2004 (n=26) [43] | - Retrospective single center chart review of hematopoietic stem cell transplantation with oliguric AKI  - RRT was initiated for patients with > or =10% FO | Cumulative fluid input & output | - Mean maximum %FB of the included patients was 9%±5% (range 3%–18%)  - All 11 survivors either maintained <10% FB during their course or re-attained <10% FB with RRT treatment |
| Goldstein, 2005 (n=116) [9] | - Prospectively collected multi-center registry data (ppCRRT)  - ≥ 2 organ dysfunction  - CRRT, <18 years old, 2001-2005 | Cumulative fluid input & output | - FB was still significantly lower for survivors versus non-survivors (14% vs 25% p < 0.05) controlling for severity of illness  - Survival rates were significantly better for patients with <20% FB versus >20% FB at CRRT initiation (58% vs 40%, p < 0.002) |
| Flores, 2008 (n=51) [44] | - Prospectively collected multi-center registry data (ppCRRT)  - CRRT after hematopoietic stem cell transplantation | Cumulative fluid input & output | - Mean %FB at CRRT initiation 12.41±3.70, Median 3.28 (-9.26-158.04)  - FB at CRRT initiation survivors 10.60±5.55 vs non-survivors 13.90±5.03, p = NS |
| Hayes, 2009  (n=76) [12] | - Retrospective, single-center  - CRRT, <18.9 years old, 2000-2005, PICU | Cumulative fluid input & output | - Median %FB at the time of CRRT initiation was lower in survivors (7.3% vs 22.3%, p = 0.0001)  - FB >20% associated with increased mortality, independent of illness severity and other clinical factors aOR 6.1, 95%CI 2.2-17, p = 0.0006)  - FB >20% also independently associated with hospital LOS, duration of mechanical ventilation, and time to renal recovery. |
| Baird, 2010 (N=39) [45] | - Single Center retrospective  - Compared long duration CRRT (>4 weeks) to short duration | Cumulative fluid input & output | - Median FB was 18% (range: 1-43%) at CRRT initiation  - Critically-ill patients treated with long and shorter duration CRRT had a similar survival rate |
| Elbahlawan, 2010 (N=30) [46] | - Single center Retrospective analysis    - Hematopoietic stem cell transplantation cohort from 1994 to 2006 who received CRRT during a course of mechanical ventilation for acute lung injury | Cumulative fluid input & output | - Improvement in PaO(2)/FiO(2) correlated significantly with reduction of FB achieved after initiation of CRRT (P = 0.0001) |
| Sutherland, 2010 (n=297) [20] | - Prospectively collected multi-center registry data (ppCRRT)  - CRRT, <18 years old, all ICU's 2001-2005 | Cumulative fluid input & output | -153 patients (51.5%) developed < 10% FB, 51 patients (17.2%) developed 10%-20% FB, and 93 patients (31.3%) developed > or = 20% FB  - FB >20% associated with increased mortality, independent of illness severity and other clinical factors (aOR 8.5)  - Adjusted OR for mortality was 1.03 (95% CI, 1.01-1.05), suggesting a 3% increase in mortality for each 1% increase in FB |
| Selewski, 2011 (n=113, 50 patients on ECMO) [17] | - Retrospective, single-center  - CRRT, all ICUs, 2006-2010 | Cumulative fluid input & output and Weight-based | - Weight-based definition of FB provided similar conclusions as fluid balance method  - Higher FB associated with increased mortality, independent of illness severity score |
| Lombel, 2012 (n=21)[31] | - Single center retrospective  - Evaluated weight-based and fluid inour & output methods of defining FO at CRRT initiation  - Hematopoietic stem cell transplantation receiving CRRT | Cumulative fluid input & output and Weight-based | - The number of patients identified as having >10% FB varied significantly according to the definition used, from 14 to 48% (p = 0.002).  - Significant intra-subject variability was observed; the median difference between individual minimum and maximum %FB scores was 11.4% (IQR 6.8, 17.1%). |
| Askenazi, 2013 (n=84) [47] | - ppCRRT registry  - Children < 10 kg | Cumulative fluid input & output | - Children ≤10 kg had lower survival rates than children >10 kg (43% vs. 64%; P < .001)  - Median FB CRRT at CRRT was lower in survivors (8% (IQR 0.36-20%) vs. 22.3% (IQR 4-44%, p = 0.03)  - Multivariable showed that FB at CRRT initiation associated with mortality aOR 4.8 comparing < 10% to > 20% |
| Boschee, 2014 (N=90) [48] | - Single center retrospective review CRRT and peritoneal dialysis in PICU, 2004-2008  - 32% ECLS | Cumulative fluid input & output | - Median FB at RRT initiation was 13.3% (IQR 4.8-22.4%),  - Median degree of FB did not differ at RRT initiation in survivors vs. non-survivors (12.1% (IQR 4.8-36) vs. 20.1% (IQR 6.4-26.8), p = .270) |
| Jhang, 2014 (n=83) [49] | - Single center PICU  - CRRT 2005-2011 | Cumulative fluid input & output | - ≥20 % FB associated with increased mortality (aOR 4.5, 95%CI 1.3–15.8, p = 0.020  - Percentage FB at CRRT initiation in the cohort 16.47 ± 21.28  - The degree of FB at CRRT initiation did not differe between survivors and non-survivors (13.10 ± 16.97 vs19.84 ± 24.61, p= 0.143) |
| Modem, 2014 (n=190), [50] | - Retrospective single center cohort study 2000-2009  - ECLS excluded | Cumulative fluid input & output | - Multivariable logistic regression analysis identified Delayed CRRT initiation (aHR 4.63, 95% CI 1.46-14.64) and FB (aHR 2.46, 1.15-5.4) as predictors of mortality  -The mean FB for nonsurvivors and survivors was 18.9% and 13.4% (median, 16.4% vs 8.8%), |
| de Galasso, 2016 (n=131)[51] | - Retrospective chart review  - 2000-2012 | Cumulative fluid input & output | - Mean FB of 7.3 % at CRRT initiation.  -At initiation of RRT, 40.5 % patients had FB>10 %  - FB>10 % at the beginning of CRRT seems to be a negative predictor of mortality (OR 10.9, 95 % CI 0.78–152.62; p=0.07) only in children with milder disease (renal patients) |
| Lee, 2016 (n=34) [52] | - Retrospective chart review of neonates on CRRT, 2007-2014  - Neonates CRRT | Cumulative fluid input & output | - Mean %FB at CRRT initiation 17.6% ± 20.8%  - Decreased survival FB > 30 % at CRRT initiation |
| Choi (n=123), 2017 [53] | - Retrospective single center PICU, “AKI requiring CRRT” | Cumulative fluid input & output  - The extent of FO 24 hours prior to CRRT initiation | - The median %FB 24 hours prior to CRRT initiation was 1.4% (0.2–3.7%)  - The median %FB 24 hours prior to CRRT  initiation differed between the survivors and the non-survivors (1.2% ± 2.2% versus 4.1% ± 4.6%, <0.001)  - Multivariable analysis %FB 24 hours prior to CRRT initiation did not predict mortality |
| Kaempfen (n=71), 2017 [54] | - Single center, PICU  - Children ≤ 10 kg initiating CRRT | Cumulative fluid input & output | - Median %FB at CVVH initiation was lower in survivors (4.26 (IQR 2–12.2) vs 11.75 (IQR 6.6–18.36), p 0.002)  - Multivariable analysis FB did not predict survival |
| Barhight, 2018 (n=66) [55] | - Retrospective cohort study, 2014-2015  - Evaluated impact of hyperchloremia on the need for RST | Cumulative fluid input & output | - Median %FB was higher at CRRT initiation in children with hyperchloremia 11.5% (IQR 3.8-22.4) vs. 5.5% (5.5% IQR 0.9-13.9) (p = 0.04)  - Multivariable analysis showed an aOR for mortality of 10.9 (95% CI 2.4 to 49.5, p = 0.002.) in children with hyperchloremia |
| Miklaszewska, 2019 (n=46)[56] | - Retrospective, single-center  - CRRT, PICU 2009-2016, | Cumulative fluid input & output | - The mortality of children with FB% ≥ 25% was more than 2-fold higher than the mortality of children with FB% < 25% 933.3% vs 67.9%, P < 0.001) |
| Cortina, 2019 (n= 161), [57] | - Retrospective study utilizing prospective registry  - Mixed population including ECMO  - Cumulative fluid balance | Cumulative fluid input & output | - Multivariable analysis predicting mortality  - FB 10%-20% (aOR 3.83; 95% CI, 1.33-11.07; p = 0.013)  - FB > 20% (aOR, 15.03; 95% CI, 4.03-56.05; p < 0.001)  - Timing of initiation of continuous renal replacement therapy (per hour, aOR, 1.01; 95% CI, 1.00-1.01; p = 0.040) |
| Chen, 2021 (n=289),  [58] | - Single-center PICU  - Children receiving 2010-2019 | Cumulative fluid input & output | - There was an association with greater survival in those with FB less than10% at CRRT initiation  ([99/124] 79.5% vs. [5/32] 15.6%, p < 0.001)  - Comparing patients 2010- 2014 vs 2015-2019: The later cohort had decreased time to initiation of CRRT and decreased %FB at CRRT initiation |
| **Neonates** |  |  |  |
| Schmidt, 2006 (n=999) [29] | - Secondary analysis of the randomized clinical Trial of Indomethacin Prophylaxis in Preterms (TIPP), multi-center  - Extremely low birthweight infants | Weight-based | - Indomethacin prophylaxis reduced urine volume during the first 4 days of life  - The average weight loss after the first postnatal week predicted the development of bronchopulmonary dysplasia |
| Askenazi, 2013 (n=58) [4] | - Prospective observational study, single-center  - Sick near-term/term neonates (> 2000grams, gestational age ≥ 34 weeks) | Weight-based | - AKI in 15.6% of the population  - Median weight change at day of life 3 was higher in those with AKI (8.2% (IQR 4.4,21.6)) than without AKI (−4% (IQR−6.5, 0.0) (p<0.001))  - Neonates with AKI had lower survival than those without AKI (72 % vs. 100 %, p<0.02) |
| Askenazi, 2015 (n=122) [59] | - Prospective cohort study  - Premature infants (birthweight ≤ 1200 grams and/or < 31 weeks gestation) | Weight-based | - AKI occurred in 30%  - Those with AKI had a higher percentage maximum weight change (max % weight Δ) in the first 4 days (RR 1.45, 95 % CI 1.07–1.97); p < 0.02). |
| Selewski, 2020 (n=1007) [18] | - AWAKEN dataset  -Preterm neonates  - Outcome: MV postnatal day 7 | Weight-based | - Multivariable models showed that the following fluid balance variables (1st postnatal week) were independently associated with the need for mechanical ventilation on postnatal day 7:  - Peak fluid balance (aOR 1.14, 95% CI 1.10-1.19)  - Lowest fluid balance (aOR 1.12, 95% CI 1.07-1.16)  - Fluid balance on postnatal day 7 (aOR 1.10, 95% CI 1.06-1.13) |
| Selewski, 2019 (N=645)[14] | - AWAKEN dataset  -Term neonates  - Outcome: MV postnatal day 7 | Weight-based | - Multivariable models showed that the following fluid balance variables (1st postnatal week) were independently associated with the need for mechanical ventilation on postnatal day 7:  - Peak fluid balance (aOR 1.12, 95%CI 1.08-1.17)  - Lowest fluid balance (aOR 1.14, 95%CI 1.07-1.22)  - Fluid balance on postnatal day 7 (aOR 1.12, 95%CI 1.07-1.17)  - Negative fluid balance on postnatal day 7 (aOR 0.3, 95%CI 0.16-0.67) |
| Matsushita, 2020 (n=219) [60] | - Single center retrospective  - BW < 1000 grams | Cumulative fluid input & output  Fluid balance over first 3 postnatal days | - Compared negative fluid balance; mild fluid overload (0- 10% of birth weight); moderate fluid overload (10 and 15% of birth weight); and severe fluid overload (fluid balance >15% of birth weight).  - Early FB>15% over the first 3 postnatal days associated with increased morality and MV |
| Rallis, 2021 (n=103) [61] | - Retrospective cohort study  - Infants ≤30 weeks' gestation and ≤1500 grams' birth weight | Cumulative fluid input & output | - Threshold ≥ 5% at the end of postnatal day 1  - 41% had FB ≥ 5%  - FB ≥ 5% associated with PDA |
| **Cardiac Surgery** |  |  |  |
| Hazle, 2013 (n=49) [13] | - Prospective observational study  -Post-operative cardiac surgery, < 6 months of age, 2009-2010 | Weight-based | - Patients with adverse outcomes had higher mean maximum FB (24±15% vs. 14±8%, p=0.02)  - Maximum FB > 10% associated with poor outcomes |
| Seguin, 2014 (n=193) [91] | - Retrospective cohort study, single-center  -Post-operative cardiac surgery, < 18 years of age,2005-2007 | Cumulative fluid input & output | - Peak FB 7.4±11.2% and occurred on post-op day 2  - Day 2 FB % predicted longer length of stay (aHR, 0.95; 95% CI, 0.92–0.99; p = 0.009) and length of ventilation (aHR, 0.97; 95% CI, 0.94–0.99; p = 0.03) |
| Hassinger, 2014 (n=98) [11] | - Secondary analysis of a prospective observational study,2009-2010  - Post-operative cardiac surgery, < 18 years of age | Cumulative fluid input & output | - FB > 5% at end of postoperative day 1 occurred in 31% of patients  - FB > 5% preceded the development of AKI  - FB > 5% associated with length of stay (3.5 days) and were more likely to require prolonged mechanical ventilation (p<0.001 |
| Sasser, 2014 (n=52) [62] | - Enrolled 52 consecutuve patients  - CPB  - Comparison of passive peritoneal drainage and diuretics (controls) versus active pd | Cumulative fluid input & output reported as mL/kg | - Median fluid balance was more negative in patients undergoing active peritoneal dialysis at 24 hours, (-24 mL/kg (IQR: -62, 11) vs. +18 mL/kg (IQR: -26, 11), P = .003)  - Median fluid balance was more negative in patients undergoing active peritoneal dialysis at 24 hours 48 hours, (-88 mL/kg (IQR: -132, -54) vs. -46 mL/kg (IQR: -84, -12), P = .004) |
| Sampaio, 2015 (N=85) [63] | - Single center retrospective chart review  June 2010 and December 2013 | Cumulative fluid input & output | - Maximum cumulative fluid balance was associated with duration of mechanical ventilation (aβ = 0.53, CI 0.38-0.66, P < 0.001) and length of stay in the pediatric intensive care unit (Spearman's correlation = 0.45, P < 0.001)  - Maximum cumulative fluid balance was correlated with maximum OI (Spearman’s test = 0.37, p = 0.01) |
| Piggot, 2015 (n=95) [64] | - Neonates undergoing cardiac surgery (<30days) | Cumulative fluid input & output | - 27% of patients had peak FB > 15% in the first 72 hours post-op  - Fluid overload (defined as > 15% in first 72 hours) and AKI led to increased LOS and MV |
| Lex, 2016 (n=1530) [37] | - Prospective cohort 2004-2008 | Cumulative fluid input & output reported as mL/kg | - 1367 patients (89.9%) had a cumulative FB < 5%; 120 patients (7.8%), between 5-10%; and 33 patients (2.1%), > 10%  - Higher FB on the day of the surgery was independently associated with mortality (aOR, 1.14; 95%CI, 1.008-1.303; p = 0.041)  - Cumulative FB on postoperative day 2 was associated with prolonged mechanical ventilation (aOR 1.012, 95% CI 1.005-1.032, p = 0.025) |
| Park, 2016 (n=) [65] | - Single center retrospective study 2012  - FO via Goldstein method | Cumulative fluid input & output | - FB during first 3 days post-op did not predict AKI (4.17% (IQR: -5.07–7.23) vs. -5.59% (IQR: -9.73–4.41) <0.001) |
| Wilder, 2016 (n=435) [66] | - Single center retrospective study  - Neonates (≤ 30 days old) cardiac surgery with CPB from January 2006 to December 2010 | Weight-based | - FB measured on POD3 was significantly higher those with “poor” composite outcome (18.6% vs 9.7%; p = 0.002)  - FB > 20% associated with poor composite outcome (Death, RST, ECMO within 30 days)  - ROC for poor composite outcome identified FBb > 16% on post-op day 3 as an important predictor |
| Kwiatkowski, 2017 (n= 73) [67] | - Single-center, unblinded, randomized clinical trial  fluid removal after cardiac surgery from October 1, 2011, through March 13, 2015  - Infants received intravenous furosemide (1mg/kg every 6 hours) or a standardized PD regimen  - Primary end point was incidence of negative fluid  balance on postoperative day 1 | Cumulative fluid input & output | - No difference was found between treatment groups in the incidence of negative fluid balance on post-op day 1  - The furosemide group was 3 times more likely to have 10% FO (OR, 3.0; 95% CI, 1.3-6.9) |
| Delpachitra, 2017 (n= 45 cases) [68] | - Case-control  - Deaths during ICU admission following cardiac surgery (cases) were matched 1:3 with children who survived to ICU discharge | Cumulative fluid input & output | - Median cumulative percent fluid balance on post-op day 2 did not predict increased mortality (1.05% (IQR:–2.25-5.88) vs 1.98% (IQR: 0.56-5.46), p = 0.65)  - Median cumulative percent fluid balance on post-op day 7 did not predict increased mortality (–7.32% (IQR: –11.79 to 2.35) vs. –10.95% (IQR: –17.91 to –2.03), p = 0.07)  - Peak serum chloride on day 2 predicted mortality  - Early FO (≥ 5% by day 2) associated with length of stay and length of mechanical ventilation |
| Soohoo, 2018 (n= 95) [69] | - Single-center retrospective, 2009 to 2015 including neonates who underwent the Norwood procedure  - Correction of serum creatinine for fo | Cumulative fluid input & output | - Correction of serum creatinine for fluid overload improves the precision of AKI diagnosis |
| Mah, 2018 (n=167) [93] | - Single center retrospective cohort study    - Neonates (age < 30 days) with cardiopulmonary bypass | Cumulative fluid input & output | - 65% developed FB > 10%  - FB > 10% was associated with mortality [aOR 1.058 (per 1% FO increase); 95%CI 1.008,1.125;p = 0.032]. |
| Bellos, 2020 (12 studies, N=3111)[24] | - Meta-analysis aimed to accumulate current literature evidence and evaluate the correlation of fluid overload degree with adverse outcome in patients undergoing congenital heart surgery |  | - Meta-analysis demonstrated a linear correlation between fluid balance:  - Mortality (χ^2^ = 6.22, p value = 0.01)  - AKI (χ^2^ = 35.84, p value < 0.001),  - Meta-analysis demonstrated a positive curvilinear relationship between fluid balance:  - Hospital LOS (χ^2^ = 18.84, p value = 0.0001)  - ICU stay (χ^2^ = 63.69, p value = 0.0001). |
| Wang, 2020 (n=88) [70] | -Retrospective study post-op repair of Anomalous origin of the left coronary artery from the pulmonary artery (ALCAPA) | Cumulative fluid input & output | - Early FO ( FB ≥ 5% at the end of post-op day 1) occurred in 37.5%  - Early FO predicted: severe AKI, longer mechanical ventilation hours (p  <  0.001), ICU LOS (p = 0.003), and hospital LOS (p = 0.009) |
| Gist, 2021 (n=41) [71] | - Single center  - Neonates undergoing arterial switch operation  - Compare peritoneal dialysis to those without | Cumulative fluid input & output | - FB in the dialysis group was significantly more negative in the dialysis group across all 7 post-op days  - PD was associated with a 42%reduction in hours of mechanical ventilation (p<.01) and a 34% reduction in ICU LOS (p=0.02) |
| Anderson, 2021 (n=70) [72] | - 2000-2012  - Children < 6yo undergoing heart transplant | Cumulative fluid input & output | - FB > 10% in the first 5 post-operative days, occurred in 23% of patients  - FB >20% was a statistically significant independent predictor of mortality (P = .005), ventilation time, and PICU length of stay. |
| Zanaboni, 2021 (n=530) [73] | - Single center retrospective review  - < 1 yo undergoing CPB  - Evaluated the impact of intra-operative ultrafiltration (UF) | Cumulative fluid input & output | - Higher total UF associated with AKI  - Higher indexed UF did not impact peak FO |
| Bailly, 2022 (n=2223) [38] and Neumayr, 2022 (n=2235) [28] | - Observational cohort study of neonates (≤ 30 days) undergoing cardiac surgery  - NEonatal and Pediatric Heart Renal  Outcomes Network (NEPHRON) study | Cumulative fluid input & output  Weight-based | - Median peak FB 4.9% (IQR 0.4%, 10.5%)  - Peak FB and postoperative day 1 FO not associated with outcomes  - Time to first day of negative fluid balance associated with outcomes (length of mechanical ventilation, ICU LOS, and Hospital LOS)  - Center variation in obtaining daily weights  - Poor correlation between fluid balance calculations and weight-based calculations |
| **General Critical Care** |  |  |  |
| Flori,, 2011 (n=313) [6] | - 2 center prospective cohort of acute lung injury | Cumulative fluid input & output reported as mL/kg/d | - Fluid balance (10 mL/kg/day increments) associated with increased mortality (aOR 1.08 (95%CI 1.01, 1.15), p = 0.02) and length of mechanical ventilation (aβ - 0.21 (95% CI -0.39, -0.04) .02) |
| Arikan, 2012 (n=80) [3] | - Retrospective, single-center  - Mechanical ventilated with arterial line at 24 hrs after PICU admission, 2004-2005 | Cumulative fluid input & output | - Peak FB was independently associated with higher peak oxygenation index, (p = .009)  - Peak FB and severe FO (≥15%) were both independently associated  - Longer duration of ventilation (p=0.008)  - pediatric intensive care unit LOS (p = .01) |
| Valentine, 2012 (n=168) [92] | - 5 PICU, MV with ALI 2007-2010, Retrospective cohort study  - Fluid balance mL/kg | Cumulative fluid input & output reported as mL/kg | - Cumulative fluid balance did not differ between survivors (80 mL/kg) and nonsurvivors (119ml/kg, p=0.11)  - Increasing cumulative fluid balance (mL/kg) on day 3 was associated with fewer ventilator-free days (p=.02)  - Daily fluid balance on days 1-3 and cumulative fluid balance on days 1-7 were higher in these children compared to adults in the Fluid and Catheter Treatment Trial conservative arm (p<.001, each day) and was similar to adults in the liberal arm |
| Abuledba, (n=317) 2014 [1] | - Retrospective analysis of multicenter (N=17) pediatric septic shock clinical and biological database | Cumulative fluid input & output | - Median %FB in first 24 hours differed between non-survivors and survivors (10.6% (IQR 3.8–15.7) vs 4.5 (IQR 1.3–9.8), p < 0.001)  - Cumulative % positive FB differed between non-survivors and survivors (19.5% (IQR 10.5-40.1) vs 6.5% (IQR -1.3-14.6), p < 0.001)  - Increased cumulative percent positive fluid balance was associated with mortality in the low-risk cohort (aOR, 1.035; 95% CI, 1.004-1.066) |
| Ketharanathan, 2014, (n=100) [96] | - Single center prospective cohort study PICU admissions Feb-Mar 2013 | Cumulative fluid input & output | - Patients who died had higher mean (IQR) FB [4.9 (2.9-9.3)% vs. 3.4 (1.9-4.8)%; p = 0.04]. |
| Bhaskar, 2015 (n=114) [5] | - Retrospective case-control  - PICU admissions with shock, ≤18 yo, 2009-2010 | Weight-based | - Early FO (FB ≥10% in 72 hours) occurred in 37% of patients  - Early FO occurred more commonly in non-survivors (73 vs. 31%, p=0.003)  - Logistic regression identified early FO as predictive of mortality (aOR 9.2, 95%CI 2.2,55.6) |
| Sinitsky,2015 (n=636) [19] | - Retrospective cohort study  - Patients invasively ventilated at 48 hours following PICU admission, age <16 years, 2009-2013 | Cumulative fluid input & output | - Median FB at 48 hours was 7.2% (IQR: 4.4,12.2)  - Linear regression showed that FO at 48 hours predicted oxygenation index and length of invasive ventilation |
| Chen, 2016 (n=202)[94] | - Single center PICU severe sepsis | Cumulative fluid input & output | - 41 (20.3%) patients experienced early FO (FB ≥5% in the first 24 hours of admission)  - Among patients with at least 48 hours of PICU stay (n = 154), 36 (23.4%) developed PICU-acquired daily FO  - Early FO (aOR = 1.20; 95% CI 1.08-1.33; P = 0.001; n = 202) and PICU-acquired daily FO (aOR = 5.47 per log increase; 95% CI 1.15-25.96; P = 0.032; n = 154) were independent risk factors associated with mortality |
| Li, 2016  (n=370) [27] | - Prospective observational study  - PICU admissions > 24 hours, Age 1 month - <16 years, 2009-2010 | Cumulative fluid input & output | - Early FO (FB ≥5% at 24 hours) developed in 17.3% of patients  - Early FO was associated with AKI  - Early FO predicted increased mortality (aOR 1.17, 95%CI 1.01,1.37, p=0.035) |
| Diaz, 2017 (n=224) [74] | - Single center prospective cohort study children MV for > 24 hours  - Daily FO was calculated as [(fluid in-fluid out)/admission weight]×100% | Cumulative fluid input & output | -Median peak FB was 12.5% (IQR 5, 25)  - Peak FB>10% was present in 55.8% and >20% was present in 33%  - Peak FB in non-survivors was 17.8% (IQR 8, 30) vs 11% (IQR 4, 23) in survivors (p=0.028)  - Survival analysis showed no association between peak FB and mortality  - Peak FB associated with length of MV and PICU LOS |
| Ingelse, 2017 (n= 132)  [75] | - Retrospective single center PICU  - MV, Age < 2 years | Cumulative fluid input & output  reported as mL/kg (reported on day 3 of MV) | - Mean cumulative fluid balance on day 3 was + 97.9 (49.2) mL/kg  - Multivariable linear regression analysis showed that higher cumulative fluid balance on day 3 was associated with a increased length of MV (β = 0.166; p = 0.048) |
| Barhight, 2018 (n=1935) [81] | - Single center, Retrospective cohort study, 2014-2015  - Evaluated impact of hyperchloremia on outcomes | Cumulative fluid input & output | - The median FB at 48 hours was 5.% (IQR 2.2-8.6)  - Multivariable analysis: increase in chloride ≥ 5 mEq/L gave aOR 2.3 (95% CI 1.03-5.21) of mortality |
| Samaddar, 2018 (n=118) [76] | - Prospective observational study  -Age 1 month-15 years critically ill mechanically ventilated | Cumulative fluid input & output | - Median FB at 48 hrs of ICU admission was higher in non-survivors (9% (6.1, 12.7) vs 6.6% (3.1, 10.3), p = 0.04)  - Cumulative fluid balance of ≥15% was observed in 74 (62.7%)  - FB > 15% associated on multivariable analysis with length of MV and ICU LOS |
| Sethi, 2018 (n= 102)[77] | - Prospective study, pediatric intensive care in a tertiary hospital. June 2013-June 2014  - Inclusion criteria: invasive mechanical ventilation for >24 h and had an indwelling arterial catheter. | Cumulative fluid input & output | - Mean maximum FO 8.7 ± 8.1%  - On adjusted analysis peak FO% was associated with increase mortality: mortality (FO <5% is reference):  - FB 5–9.99% aOR 2.520 (1.80–7.92),  - FB 10–14.99% aOR 2.751 (1.32–15.66)  - FB >15% aOR 3.675 (1.28–23.18) 0.039 |
| Muttath, 2019 (n=291) [78] | - Prospective observational study  - 1 month-16 years with shock requiring inotropes and/or mechanically ventilated | Cumulative fluid input & output and mL/kg | - FB higher in non-survivors at 72 hours of admission (13.95 ± 9.6 mL/kg in non-survivors vs 10.97 ± 6.4 mL/kg in survivors; *p* = 0.022) at 72 hours  - A higher mortality was observed in children with higher PFO (>20% FO: 45.8% mortality vs. 14.5% < 10% FO, *p*  < 0.01)  Cox analysis: FB predicted increased length of LOS, increase OI |
| Vaewpanich, 2019 (42 cases) [79] | - Case control study  - Evaluated the impact of fluid overload on the development of ventilator associated conditions (VAC) | Cumulative fluid input & output | - Individuals with VAC had higher peak daily FB% within 3 preceding days (mean (SD), 8.1(7.8) vs. 4.1 (3.4), p < 0.005)  - Multivariable analysis showed that the FOKIS score predicted VAC |
| Gist, 2020 (n=149) [95] | - Single-center quaternary level PICU. Secondary analysis of prospective cohort study | Cumulative fluid input & output | - 24.2% had FB > 20% on day 3 of ICU admission  - Evaluated phenotypes AKI, FO:  - FO^+^/AKI^+^ patients had longer LOS on multivariable analysis (17.4 (11.0–23.7; 95%CI) vs. 8.8 (7.3–10.9; 95%CI), p=0.05)  - Correction of AKI for fluid balance delineated significant change in class switching of AKI stage in 29 patients |
| Al-Lawati, 2020, (n=102), [97] | - Single center prospective observational cohort study  - PICU Admitted for > 24 hours | Cumulative fluid input & output | - Of patients admitted for 3 days (n=69) 43% developed FO at a median of ICU day 2.5 (IQR, 1-4)  - FO was only documented in 23% of these cases |
| Alobaidi, 2020 (n=1017), [2] | - Retrospective cohort study  - Provincial PICU dataset (Alberta, Canada)  - All admission 2015 | Cumulative fluid input & output  Threshold for FO ≥ 15%  Evaluated trajectory | - Proportion of patients with peak FB > 10% was 32.7% (29.8-35.7%) and > 20% was 9.1% (7.4-  11.1%)  - Peak FB was associated with greater PICU mortality (odds ratio, 1.05; 95% CI, 1.02-1.09; p = 0.001)  - Greater peak fluid overload % was associated with:  - Major Adverse Kidney Events within 30 days (odds ratio, 1.05; 95% CI, 1.02-1.08; p = 0.001)  - Length of mechanical ventilation (B coefficient, 0.66; 95% CI, 0.54-0.77; p < 0.001)  - Length of PICU stay (B coefficient, 0.52; 95% CI, 0.46-0.58; p < 0.001)  - The rate of fluid accumulation was associated with adverse outcomes |
| Barhight, 2020 (n=348) [80] | - Evaluated those with AKI on admission day 2  - Evaluated the impact of hyperchloremia on renal recovery | Cumulative fluid input & output | - Those with persistent hyperchloremia (high chloride on day 2 and 7 had worse outcomes relative to those with normalized Cl  - lower odds of day 7 renal recovery (aOR = 0.29; 95% CI, 0.14 to 0.60; p = 0.0009),  - lower odds of discharge renal recovery (aOR = 0.22; 95% CI, 0.11 to 0.48; p = 0.0001)  - higher odds of mortality (aOR = 3.50; 95% CI, 1.11 to 11.10; p = 0.03) |
| Lima, 2021 (n= 177) [30] | - Single center prospective cohort study  - Children 3 months to 25 years old admitted to the PICU with expected length of stay greater than or equal to 48 hours | Cumulative fluid input & output | - Peak FB occurring > 48 hours of ICU admission was associated with:  - Longer median ICU LOS (8 (IQR4-15) vs 4 d (IQR 3-8 d); p ≤ 0.001]  - Hospital LOS (18 [10-38) vs 12 [8-24]; p = 0.01)  - Increased risk of mortality (n = 10 [13%] vs 2 [2%]; χ2 = 7.6; p = 0.006].  - Timing of negative fluid balance (Day 3-7) was also associated with increased length of MV, ICU LOS, and hospital LOS. |
| Black, 2021 (n=723) [82] | - Single center  - Intubated children | Cumulative fluid input & output reported as Cumulative Fluid Balance (ml/kg) and  Daily Fluid Balance (mL/kg) | - Late FO (after day 4 of ARDS onset), associated with increased PICU mortality and fewer ventilator free days |
| Rameshkumar, 2021  (n= 888) [83] | - prospective cohort study,  - children < 13  - admitted > 48 h | - Weight-based | - Compared those with positive fluid vs negative fluid balance for first 7 days of ICU admission  - Positive fluid balance associated with increased length of stay (ICU and Hospital) |
| Armenda, 2021  (n=146) [84] | - Single center Retrospective  - January 2016 to May 2020  - Dengue Shock Syndrome | - Cumulative fluid input & output | - FB ≤ 10% (0% mortality) vs > 10% (15% mortality)  - Fluid balance percentage, shock condition at PICU admission, DIC, and AKI were independent predictors for DSS mortality |
| **ECMO** |  |  |  |
| Hoover, 2008 (n=52) [85] | - Retrospective case-matched study (CRRT + ECMO vs. ECMO alone)  - All pediatric patients receiving respiratory ECMO, Age 1mo-18 years, PICU, 1992-2006 | Cumulative fluid input & output reported as mL/kg/day | Survivors 25ml/kg/d vs. nonsurvivors 40ml/kg/d  - Survival was not significantly different those on CRRT  - Use of CRRT with ECMO was associated with improved fluid balance, improved caloric intake and decreased diuretic exposure |
| Blijdorp, 2009 (n=61) [86] | - Retrospective case-comparison study  - Pre-emptive CVVH during ECMO, age <28 days | Cumulative fluid input & output | - Those treated with CRRT had shorter median duration of ECMO (98 hrs (IQR48,178) vs 126 hrs (IQR 24,403))  - Those treated with CRRT had a shorter duration of intubation following ECMO decannulation and required less blood transfusions |
| Selewski, 2012  (n=53) [16] | - Retrospective, single-center  - CRRT, all patients treated with CRRT and ECMO, 2006-2010 | Weight-based  Continuous and  Categorical | - Median FB at CRRT initiation was lower in survivors (24.5% vs 38%, p=0.006)  - Median FB at CRRT discontinuation was lower in survivors (7.1% vs 17.5%, p=0.035)  - Models evaluating fluid removal consistently showed that the degree of FB at CRRT initiation predicted mortality |
| Murphy, 2018 (n=63) [87, 88] | - Retrospective before after study  - Neonate on ECMO  - Intervention was institution of protocol for early CRRT initiation for neonates on ECMO | Weight-based | - Decreased weight gain at CRRT initiation and faster return to baseline weight during the first 7 days of ECLS  - Early CRRT population received better nutrition |
| Selewski, 2017 (6 centers, n=756) [15] | - Retrospective chart review of all children < 18 years treated with ECMO, 2006-2010) | Cumulative fluid input & output | - Median peak FB on ECMO was 30.9% (IQR, 15.4,54.8)  - Peak FB during ECMO: 84.8% ≥ 10%; 67.2% ≥20% and 29% ≥50%.  - Multivariable analysis showed that multiple measures of FB were associated with outcomes:  - peak FB on ECMO (adjusted odds ratio, 1.09; 95% CI, 1.04-1.15) predicted mortality on ECMO  - FO at ECMO initiation (aOR, 1.13; 95% CI, 1.05-1.22) and peak FB (aOR, 1.18; 95% CI, 1.12-1.24) both predicted hospital morality. |
| Gorga, 2020  (n= 756), [10] | - Retrospective chart review of all children < 18 years of age concurrently treated with **ECMO and CRRT** six tertiary care children's hospital | Cumulative fluid input & output | - Median FB at CRRT initiation was 20.1% (IQR 5, 40) and was significantly lower in ECMO survivors vs. non-survivors (15.3% vs. 30.5% p = 0.005) and in hospital survivors vs. non-survivors (13.5% vs. 25.9%, p = 0.004)  - Multivariable analysis, FB at CRRT initiation (aOR 1.09, 95% CI 1.00-1.18, p = 0.045) and at CRRT discontinuation (aOR 1.11, 95% CI 1.03-1.19, p = 0.01) were independently associated with hospital mortality |
| Mallory, 2020 (n= 424), [89] | Retrospective chart review of all children < 18 years of age concurrently treated with six tertiary care children's hospital  - Evaluated the impact of AKI and FO in those with Respiratory indication for ECMO | Cumulative fluid input & output | - Peak FB nonsurvivors 37% vs. survivors 30%,  - For every 10% increase in peak FB during ECMO, the odds of hospital mortality were approximately 1.2 times higher.  - Every 10% increase in peak FB during ECMO resulted in a significant relative change in the duration of ECMO hours by a factor of 1.08.  - For hospital survivors, every 10% increase in peak FB level during ECMO resulted in a significant relative change in the duration of mechanical ventilation hours by a factor of 1.13. |
